# Lunacy

**Published:** 1859-07

**Authors:** 


### LUNACY.

Of all the assigned causes of lunacy in the Pennsylvania Asylum, nearly one out of every four, arose from "domestic trouble," the women numbering one-third more than the men.

This ought not to be, for the nearest approach to human happiness, other things being equal, is in having a family, and a home, where affection, love, and sympathy find daily food, and grow and expand in buds and blossoms, and luscious fruit. The family fireside, the family table, these make the earthly heaven of us all; these yield the oil which calms the troubled waters of business life; the springs whose crystal draughts allay the feverish excitements, and the burning thirst created by worldly turmoil; these are they which send off sweet perfumes, which purify the moral atmosphere, and feed the dearest affections of our nature.

Why should a road ever end in a mad house, which ought always to terminate in an earthly paradise? Surely an asylum is not the natural *finale* of the marriage ceremony; but that it is becoming oftener so than in past years, seems to be a conceded fact. That marriage does not of itself tend to madness, appears to derive confirmation in the fact, that there are more single, than married persons in our lunatic asylums. The difference however is a mere fraction, it ought to be wide as the poles asunder. Why then are there so many more married persons in our mad houses than there ought to be, considering the greater number of resources for happiness which belong to the married?

If a married man becomes deranged, we will venture the assertion, without any undue prejudice against women, that in numerous cases, it largely is the fault of his wife; although we are free to confess, that a man cannot be at a great remove from daftness, who will go crazy from any of the ordinary causes of domestic disquietude; yet a man, or woman either, may so nurse a very insignificant matter, by brooding over it, as to make it enlarge to such an extent, that it will literally shut out from the vision, the universe besides, and then, the mind is a blank.

The prevailing weakness of the women of our time, especially in cities, is their passion for dress and climbing. It is useless to spend time and paper, in proving this, the observant are conscious of the fact. In reference to their wives, men are weak. The sternest philosophy of the office and the counting room, is demolished at a stroke, at "the house." Most men love to see their wives and daughters well dressed, however slouchy they may go themselves, and between this pride and the desire to gratify their families, as well as to save the trouble of a firm resistance, and the inevitable consequences of that resistance, the fortress is surrendered; involvements follow, struggles succeed, and failure, despondency and despair, come on apace, and the grave or grated door, shut out the sun light of life forever.

Next to this, is the passion for climbing; that jackal of unrest, that will have food, even if it be dead men's bones and carcases, only if it afford the aliment of better appearances, the capability of taking another step higher, in order to reach a "set"

beyond. Men are too much absorbed in business, to care about such things; their minds are taken up with more tangible matters; with the necessities of house, and food, and clothing, and for the reasons named as to dress, yield, when they ought not to; yield for the sake of peace.

On the other hand, the wife at home, not having the mind carried off to more imperative callings, and being more at leisure to cherish trifles, allows herself to reflect on her position, and the more elevated one of others, who are, may be, not as good as she is, and are really, in many instances, not as deserving. This causes at length a disquiet, a dissatisfaction, an irritation, a settled annoyance, which is daily fed by the many little causes of fretfulness which are incident to every household, in the carelessness of domestics, their ignorance, their utter want of interest in the concerns of their employers, and their great destitution of moral principle; these are the things which wear out the lives of many excellent women, and waste them into the grave, long before their prime, or shut them in a cell.

Is there a remedy for these sad things? and if so, what is it? or if more than one, what are they?

Our daughters must be educated differently. They must be made better acquainted with the real world around them, by being made to live in the midst of fact, rather than to luxurate in the realms of fiction. They must be induced to read the Bible more, and the newspaper and the novel less. They must be taught to feel that they are neither puppets nor bandboxes, nor dolls. That there are stern duties to be performed in life, and that they are to perform them. That there is a space between a wish and its gratification. That efforts are to be made; self denials are to be practised; appetites to be curbed; passions subdued; desires mortified, and feelings restrained. They must be made to know they are not always to be served; that the time will come when they must serve others; that they must care, and work, and toil for others, as others have cared, and worked and toiled for them; that this is a world of reciprocities, and that those who practice them most assiduously, will be the happiest, the most loved, and the most admired. Let them grow up to feel that the best type of a true womanhood, is not in the cut of the dress, the style of the bonnet, the softness of the hands, the beauty of the face, the depth of the intellect, the extent of the acquisitions, whether as to music, or languages, or step, or position; not in one or all of these is there a true womanhood, but rather in the purity of the heart, in the warmth of the affections, in the abnegation of self, in the consciousness of just purposes, and in the possession of that personal dignity, which is inseparable from the qualities named.

And both as to sons and daughters, we must sedulously educate them, and that right early, in the practice and appreciation of industry, of economy, self reliance, by learning them to help themselves; in that feeling of independence, and personal self-respect, which never fails to wait on a man or woman, who has been trained to rely on their own resources, to never attempt to accomplish for themselves individually, what they cannot do without the aid of other's work, or other's wealth, or without unwise risks.

